# One‐Year Outcome of Vitreous Cortex Remnants Removal Using a Nitinol Flex Loop for Recurrent Retinal Detachment Patients

**DOI:** 10.1155/joph/6880659

**Published:** 2026-08-06

**Authors:** Zhan Xie, Yifan Lin, Haiyue Xie, Mulong Du, Hongfei Xu, Ping Xie

**Affiliations:** ^1^ Department of Ophthalmology, The First Affiliated Hospital of Nanjing Medical University, Nanjing 210029, Jiangsu, China, njmu.edu.cn; ^2^ Department of Biostatistics, School of Public Health, Nanjing Medical University, Nanjing 210029, Jiangsu, China, njmu.edu.cn; ^3^ Department of Ophthalmology, Jurong Hospital Affiliated to Jiangsu University, Zhenjiang 212400, China

**Keywords:** FINESSE Flex loop, membrane scraper, pars plana vitrectomy, recurrent retinal detachment, vitreous cortex

## Abstract

**Purpose:**

To evaluate the 1‐year outcome of vitreous cortex remnants (VCRs) removal using a nitinol flex loop during pars plana vitrectomy (PPV) in the treatment of recurrent retinal detachment (rRD).

**Patients and Methods:**

A total of 95 eyes from 95 patients with rRD with the intraoperative evidence of VCRs were retrospectively analyzed at the First Affiliated Hospital of Nanjing Medical University. Vitreous visualization was enhanced with triamcinolone. The presence of VCRs was determined by gently scraping the retinal surface with a disposable nitinol loop. Patients were divided into two groups: conventional PPV with the aid of vitrectomy probe and intraocular forceps between January 2022 and December 2022 (Group A, 41 eyes), and PPV assisted by the FINESSE Flex loop for VCRs removal between January 2023 and December 2023 (Group B, 54 eyes). Outcomes were assessed during follow‐ups at postoperative 1 week, 1 month, 3 months, 6 months, and 12 months. Primary outcomes included vitrectomy duration, single‐surgery anatomic success rate, intraoperative and postoperative complications, and best‐corrected visual acuity (BCVA).

**Results:**

Group B had a significantly shorter vitrectomy duration compared to Group A (*p* < 0.001). The incidence of intraoperative iatrogenic retinal breaks was significantly lower in Group B (3.7%) than in Group A (22.0%) (*p* = 0.015). Other intraoperative complications, including focal retinal hemorrhage and inadvertent lens touch, were comparable between groups (all *p* > 0.05). No cases of iatrogenic rhegmatogenous RD or vitreous hemorrhage were observed in either group. The single‐surgery anatomic success rate was higher in Group B (98.1%) than in Group A (85.4%) (*p* = 0.049). No significant differences were found in the total number of RD surgeries or final BCVA between groups (*p* = 0.076 and *p* = 0.109, respectively). During the follow‐up, unattached retinas occurred in 6 eyes in Group A and 1 eye in Group B; ocular hypertension was noted in 2 eyes in Group A and 3 in Group B.

**Conclusion:**

Use of the FINESSE Flex loop during PPV for rRD allows a shorter surgical duration, a lower incidence of iatrogenic retinal breaks, and a higher single‐surgery anatomic success rate, without increasing postoperative complications.

## 1. Introduction

Rhegmatogenous retinal detachment (RRD) is one of the most common ophthalmic emergencies and a leading cause of acute vision loss [1]. Proliferative vitreoretinopathy (PVR), the primary cause of failure after initial RRD repair, features the formation of preretinal contractile fibrocellular membranes and causes retinal hole formation or reopening and recurrent retinal detachment (rRD) [2]. The formation of PVR engages the intravitreal dispersion of retinal pigment epithelial cells, breakdown of the blood–ocular barrier, and retinal hypoxia; these changes are attributed to various factors, including persistent large‐extent retinal detachment, extensive large‐size retinal tears, and vitreous hemorrhage or inflammation.

Currently, PVR management merely depends on surgical repair of the redetached retina, but its effectiveness in restoring anatomical and functional integrity remains unsatisfactory. Despite significant advances in vitreoretinal surgery techniques, the incidence of PVR has stayed as high as 10–15% over the last few decades [3]. Thus, a deeper exploration into PVR pathophysiology is expected to provide information for timely therapeutic intervention and subsequent prevention of recurrence following primary RRD repair.

Notably, vitreoschisis‐induced vitreous cortex remnants (VCRs) have been reported as a common, but not sufficiently recognized, factor in the development of preretinal PVR [4]. PVR formation involves retinal ischemia, photoreceptor apoptosis, and subsequent formation and contraction of fibrotic membranes. VCRs can act as a scaffold for fibrotic cellular proliferation. Both ischemia, apoptosis, and the primary injury or retinal tear are associated with the release of proinflammatory, fibrogenic, and mitogenic molecules, which can signal to regulate the proliferation of retinal pigment epithelial cells, their epithelial‐mesenchymal transition into fibroblasts, and the deposition of collagen and extracellular matrix [5]. In addition, the hyalocytes in VCRs also play a role in the inflammatory response and PVR formation, as they can differentiate into myofibroblasts, an essential component in fibrotic contractile membranes [6]. Thus, we hypothesized that removal of VCRs during surgery may benefit RRD patients with multiple PVR risk factors, such as rRD.

In this study, we performed a retrospective single‐surgeon study of 95 eyes with rRD with the intraoperative evidence of VCRs, to explore the benefit of VCRs removal in rRD patients. Additionally, we employed the FINESSE Flex loop for VCRs removal during pars plana vitrectomy (PPV) and evaluated the clinical outcomes of surgical efficiency and anatomic success improved by this novel technique. Our results may provide evidence for designing new strategies to improve the surgical outcomes in rRD patients.

## 2. Materials and Methods

This retrospective study included 95 eyes from 95 patients with rRD who underwent PPV at the First Affiliated Hospital of Nanjing Medical University, China, between January 2022 and December 2023. Patients were divided into two groups: 41 eyes underwent conventional PPV with the aid of vitrectomy probe and intraocular forceps for VCRs removal (Group A) between January 2022 and December 2022, and 54 eyes underwent PPV assisted by the FINESSE Flex loop for VCRs removal (Group B) between January 2023 and December 2023.

### 2.1. Inclusion Criteria

Eligible patients met all of the following criteria.

1. Ages ≥ 18 years, with the ability to provide written informed consent;
2. Eyes with rRD after the failure of initial retinal detachment repair (pneumatic retinopexy, scleral buckle, and/or PPV);
3. Eyes to undergo PPV with the intraoperative evidence of VCRs;
4. The presence of VCRs confirmed by aspirating the triamcinolone particles sedimented on the retinal surface with the back flush soft tip (meanwhile, the attachment of these particles to VCRs was confirmed);
5. Sufficient corneal clarity (complete or central corneal clarity) to permit postoperative fundus examination, visual function assessment, and optical coherence tomography (OCT).

### 2.2. Exclusion Criteria

Patients were excluded if they met any of the following conditions.

1. Corneal pathology significantly impairing the visual field, such as corneal opacity or scarring;
2. Inability to maintain postoperative positioning;
3. The presence of severe ocular comorbidities, including active uveitis, uncontrolled glaucoma, or proliferative diabetic retinopathy;
4. Ongoing external ocular infection;
5. Follow‐up duration shorter than 12 months;
6. Uncontrolled systemic or ocular disease.

### 2.3. Surgical Technique

All surgeries were performed using a 23‐gauge PPV system (Constellation Vision System; Alcon Laboratories, Fort Worth, TX) in combination with a noncontact wide‐angle viewing system (Resight; Carl Zeiss Meditec, Oberkochen, Germany). Posterior vitreous detachment (PVD) was induced using the standard surgical techniques. Following core vitrectomy, a suspension of triamcinolone acetonide (4 mg/0.1 mL; Kunming Jida Pharmaceutical Co., Ltd., Kunming, China) was injected intravitreally to facilitate visualization of the posterior hyaloid. The FINESSE Flex loop was introduced through a 23G trocar. The scraper was maneuvered tangentially across the retinal surface to generate shear traction. The barbed edge was used to gently lift the vitreous cortex until a visible separation appeared between the vitreous and retina. Along this dissection plane, the bent loop was employed to further elevate the cortical remnant, which was subsequently excised using the vitrectomy probe under active aspiration. Residual vitreous cortex stained with triamcinolone was further cleared by sweeping the loop along the retinal plane in the direction of its arc. For densely adherent fibrotic strands, the loop was reversely moved to peel the membrane in the opposite direction.

### 2.4. Observational Indexes

Patients were followed postoperatively at 1 week, 1 month, 3 months, 6 months, and 12 months. Primary outcomes were measured according to vitrectomy duration, single‐surgery anatomic success rate, intraoperative and postoperative complication rates, and best corrected visual acuity (BCVA).

### 2.5. Statistical Analysis

All statistical analyses were performed using SPSS Version 21.0 (IBM Corp., Armonk, NY). Continuous variables were expressed as mean ± standard deviation (SD), median, and range and categorical variables as counts and percentages. Between‐group comparisons were made using Student′s *t*‐test for continuous variables and chi‐square or Fisher’s exact test for categorical variables. Statistical significance was defined as *p* value < 0.05.

## 3. Results

A total of 95 eyes from 95 patients with rRD were included. Among them, 41 eyes (43.2%) underwent PPV with the aid of vitrectomy probes and intraocular forceps for VCRs removal (Group A), and 54 eyes (56.8%) received PPV with FINESSE Flex loop assistance for VCRs removal (Group B). Baseline demographic and clinical characteristics were comparable between the two groups, including sex, laterality, lens status, preoperative BCVA, PVR grade [7], and surgical approach (all *p* > 0.05), as shown in Table 1.

**TABLE 1:** The basic data of patients in two groups.

Parameter	Group A	Group B	*p* value
No. of eyes/No. of patients	41/41	54/54	
Age (yrs) (mean ± SD)	55.12 ± 12.27	57.78 ± 11.77	0.288^∗^
Gender, No. (%)			0.767^†^
Men	21 (51.2)	26 (48.1)	
Women	20 (48.8)	28 (51.9)	
Eye, No. (%)			0.964^†^
Right	15 (36.6)	20 (37.0)	
Left	26 (63.4)	34 (63.0)	
Preoperative BCVA, LogMAR (mean ± SD)	2.92 ± 1.00	2.49 ± 1.10	0.052^∗^
Lens status, No. (%)			0.065^†^
PIOL	5 (12.2)	15 (27.8)	
Phakic eye	36 (87.8)	39 (72.2)	
Aphalia	0	0	
PVR grade, No. (%)			0.897^†^
B	12 (29.3)	14 (25.9)	
C1	14 (34.1)	16 (29.6)	
C2	10 (24.4)	16 (29.6)	
C3	5 (12.2)	8 (14.8)	
Surgical procedure, no. (%)			0.315^†^
PPV	24 (58.5)	37 (68.5)	
PPV + Phaco	17 (41.5)	17 (31.5)	

*Note*: Data were expressed as mean ± SD; PIOL, phakic intraocular lens; PVR, proliferative vitreoretinopathy; Phaco, phacoemulsification.

Abbreviations: BCVA, best‐corrected visual acuity; PPV, pars plana vitrectomy; SD, standard deviation.

^∗^Student’s *t*‐test analysis.

^†^Chi‐square analysis.

### 3.1. Vitreous Cutting Time and Intraoperative Complications

The mean duration of vitrectomy was significantly shorter in Group B than in Group A (*p* < 0.001), as shown in Table 2. Intraoperatively, iatrogenic retinal breaks occurred in 9 eyes (22.0%) in Group A, compared to 2 eyes (3.7%) in Group B (*p* = 0.015). The incidence of focal retinal hemorrhage was similar between the groups (17.1% in Group A vs. 5.6% in Group B, *p* = 0.140). Inadvertent lens touch was observed in 3 cases in Group A and none in Group B (*p* = 0.153). No cases of iatrogenic RRD or intraoperative vitreous hemorrhage were reported in either group.

**TABLE 2:** Vitreous cutting time and intraoperative complication profile of two groups.

Parameter	Group A (*n* = 41)	Group B (*n* = 54)	*p* value
Duration of vitrectomy (min)	23.46 ± 5.26	18.54 ± 3.36	< 0.001^∗^
Intraoperative complications, no. (%)			
Latrogenic retinal break	9 (22.0)	2 (3.7)	0.015^†^
Focal retinal hemorrhage	7 (17.1)	3 (5.6)	0.140^†^
Inadvertent lens touching	3 (7.3)	0	0.153^†^
Latrogenic RRD	0	0	—
Vitreous hemorrhage	0	0	—

*Note:* Data were expressed as mean ± SD; Min, minute.

Abbreviations: RRD, rhegmatogenous retinal detachment; SD, standard deviation.

^∗^Student’s *t*‐test analysis.

^†^Chi‐square analysis.

### 3.2. Anatomic Outcomes and Postoperative Complications

The single‐surgery anatomic success rate was significantly higher in Group B (98.1%) than in Group A (85.4%) (*p* = 0.049), as shown in Table 3. No significant differences were observed between the groups in the number of surgeries required for retinal detachment repair or final postoperative BCVA (*p* = 0.076 and *p* = 0.109, respectively). Final retinal reattachment was achieved in all the eyes of both groups. During the follow‐up period, unattached retinas occurred in 6 eyes in Group A (3 eyes with PVR‐related recurrence and 3 eyes with new‐onset RRD), compared to 1 eye with new‐onset RRD in Group B. Ocular hypertension developed in 2 eyes in Group A and 3 eyes in Group B Table 3.

**TABLE 3:** Anatomic outcomes and postoperative complications of two groups.

Parameter, no. (%)	Group A (*n* = 41)	Group B (*n* = 54)	*p* value
Anatomic outcomes			
Single‐surgery anatomic success	35 (85.4)	53 (98.1)	0.049^†^
Number of surgeries for RRD	1.17 ± 0.44	1.04 ± 0.19	0.076^∗^
Final retinal reattachment	41 (100)	54 (100)	—
Postoperative BCVA, LogMAR (mean ± SD)	1.63 ± 1.05	1.28 ± 1.03	0.109^∗^
Postoperative complications			
Recurrent PVR‐related RRD	3 (7.3)	0	0.153^†^
New‐onset RRD	3 (7.3)	1 (1.9)	0.425^†^
Ocular hypertension	2 (4.9)	3 (5.6)	1^†^
Macular hole	0	0	—
Macular epiretinal membrane	0	0	—
Vitreous hemorrhage	0	0	—
Endophthalmitis	0	0	—

*Note:* Data were expressed as mean ± SD. PVR, proliferative retinopathy.

Abbreviations: RRD, rhegmatogenous retinal detachment; SD, standard deviation.

^†^Chi‐square analysis.

^∗^Student’s *t*‐test analysis.

## 4. Discussion

Complete removal of residual vitreous cortex is a critical step in PPV to achieve optimal therapeutic outcomes and minimize postoperative complications [8]. Lifting a flap of VCRs on the retinal surface is a key surgical step. A direct grasp of VCRs with microforceps is challenging due to the very thin membrane‐like tissue, thus limiting the efficiency of removing VCRs. Multiple instruments and techniques such as gentle scraping and rubbing allow the creation of a breakthrough for the removal of the posterior vitreous cortex. Thirdly, this is a time‐consuming operation that requires meticulous handling; otherwise, it may cause iatrogenic injuries such as retinal holes. How to perform an efficient and safe clearance of VCRs remains a key challenge. In this study, we first reported the application of the FINESSE Flex loop for VCRs removal during PPV in rRD patients. Our findings demonstrate that the FINESSE Flex loop enhances surgical efficiency, reduces intraoperative complications, and improves prognostic outcomes in patients with rRD.

Several techniques have been proposed for the removal of VCRs, including the use of vitrectomy probes, retinal forceps, diamond‐dusted membrane scrapers, vitreous wiping with a rectangular piece of polyvinyl alcohol held with intraocular forceps, a hook made by a syringe needle, and manually fashioned loops made from 5‐0 polypropylene sutures [8–11]. These methods, however, often require sclerotomy enlargement or specialized materials that may not be readily available, or manual fashioning [10]. Furthermore, they carry an increased risk of incomplete removal of VCRs removal, particularly those in eyes with detached, thin, or atrophic retina. Thus, there remains a clinical need for safer, more efficient, and technically accessible tools to facilitate thorough VCR clearance and reduce surgical complications and RD recurrence.

**FIGURE 1:**
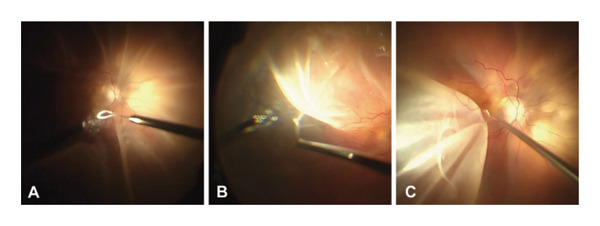
Surgical procedures using the FINESSE Flex loop to assist in removing the vitreous cortex remnants. (A) The 23‐gauge FINESSE Flex loop. The curved nitinol loop is designed to optimize scraping force, and the loop can be fully or partially retracted to modulate the pressure applied to the retina as needed. (B) The entire triamcinolone acetonide‐stained residual vitreous cortex is gently scraped from the retinal surface, following the direction of the unfolding scraper loop. (C) By reversing the sleeve of the membrane scraper, tightly adherent cord‐like membranes on the retinal surface can be peeled in the opposite direction.

The FINESSE Flex loop (Alcon, Fort Worth, TX), a flexible nitinol membrane scraper [12, 13], can be utilized for internal limiting membrane (ILM) and ERM peeling [12], stabilization of detached retina during PPV [14], intraocular lens (IOL) repositioning [12], and manual removal of silicone oil droplets or calcifications [15]. Its loop design, featuring small atraumatic tines and adjustable stiffness, allows controlled manipulation near the retinal surface [16]. The large contact area between the scraper and retina enables efficient removal of adherent VCRs while minimizing focal stress. In this study, we employed the FINESSE Flex loop for residual vitreous cortex removal during PPV for rRD. Our research proved the excellent performance of this novel surgical technique in reducing vitrectomy duration and lowering the incidence of iatrogenic retinal breaks, as shown in Table 2. This may be attributed to the soft nitinol structure and wide contact surface of the loop, which allow for gentle but effective traction along the retinal plane. Compared with vitrectomy probes and intraocular forceps, the membrane scraper demonstrated superior efficacy and safety in lifting a vitreous flap and dislodging tenacious cortical adhesions (Figure 1a,b), particularly in cases of vitreoschisis or tightly adherent posterior hyaloid. Compared with conventional diamond‐dusted brushes or membrane scrapers, the FINESSE Flex loop offers distinct structural advantages. Its nickel–titanium alloy loop avoids the risk of particle shedding or coating detachment and provides a more uniform scraping profile. During VCR removal, the loop can be gently applied to the retinal surface in a controlled direction. If a greater mechanical force is needed, the surgeon may shorten the loop arc or reverse the direction of scraping (Figure 1c). Notably, the FINESSE Flex loop employs a soft nitinol wire loop with fine tines designed for delicate membrane engagement [17]. However, in the presence of dense fibrous or proliferative membranes, an adjunctive use of intraocular forceps remains necessary.

Our cohort was composed of patients with rRD, a special type of RD carrying a high risk of PVR [18]. This population is more likely to present a wider range of tightly adherent VCRs and proliferative membranes [19, 20]. As VCRs across the retinal surface posterior to the vitreous base, due to vitreoschisis, may scaffold the development of PVR, the removal of VCRs during surgery may reduce the risk of postoperative PVR and retinal redetachment [19, 21]. Our study confirmed the efficacy of the FINESSE Flex loop in increasing the single‐surgery anatomic success in rRD, as shown in Table 3. We speculate that, compared to the vitrectomy probes and intraocular forceps in the control group, the membrane scraper increased the efficiency and thoroughness of VCRs removal in rRD patients, thereby reducing iatrogenic damage and improving outcomes.

This study has several limitations. First, it was a single‐center, retrospective analysis with a relatively short follow‐up period. A rigorously designed, multicenter randomized controlled trial with extended follow‐up is warranted to validate the long‐term efficacy and safety of this novel technique. Second, the patient cohort was only limited to rRD cases. Future studies should explore the broader applicability of the FINESSE Flex loop in other vitreoretinal pathologies, such as advanced diabetic retinopathy. It would also be helpful to further explore the extent of VCRs to be removed. It may be preferable to remove the VCRs from the macula and lower quadrants, where PVR and redetachments develop most frequently. In addition, further investigations using advanced imaging modalities, such as OCT, are needed to evaluate intraoperative and postoperative retinal microstructural changes following membrane scraper–assisted surgery.

## 5. Conclusion

The FINESSE Flex loop demonstrates favorable 1‐year clinical outcomes in the surgical management of rRD. Its application facilitates efficient and complete removal of VCRs, reduces vitrectomy duration and the incidence of iatrogenic retinal breaks, and increases single‐operation retinal reattachment rates. This technique offers a promising adjunct in vitreoretinal surgery and warrants further validation in larger, diverse patient populations.

## Author Contributions

Zhan Xie, Hongfei Xu, and Ping Xie designed the research project. Zhan Xie, Yifan Lin, and Haiyue Xie performed the experiments. Zhan Xie and Mulong Du analyzed the data. Zhan Xie drafted the manuscript. Hongfei Xu and Ping Xie reviewed and revised the manuscript.

## Funding

This study was supported by a grant from the National Natural Science Foundation of China (Grant Number: 82401281, 82371076).

## Ethics Statement

This retrospective study was approved by the Ethics Committee of the First Affiliated Hospital of Nanjing Medical University (Approval No. 2025‐SR‐367) and was conducted in accordance with the Declaration of Helsinki. Written informed consent was obtained from all patients prior to surgery.

## Consent

Please see the Ethics Statement.

## Conflicts of Interest

The authors declare no conflicts of interest.

## Data Availability

The data that support the findings of this study are available from the corresponding authors upon reasonable request.
